# Point-of-Care Tests for HIV Drug Resistance Monitoring: Advances and Potentials

**DOI:** 10.3390/pathogens11070724

**Published:** 2022-06-25

**Authors:** Rayeil J. Chua, Rupert Capiña, Hezhao Ji

**Affiliations:** 1 National Microbiology Laboratory at JC Wilt Infectious Diseases Research Centre, Public Health Agency of Canada, Winnipeg, MB R3E 3R2, Canada; rayeil.chua@phac-aspc.gc.ca (R.J.C.); rupert.capina@phac-aspc.gc.ca (R.C.); 2 Max Rady College of Medicine, University of Manitoba, Winnipeg, MB R3E 0J9, Canada

**Keywords:** HIV, drug resistance, point-of-care test, resource-limited setting

## Abstract

HIV/AIDS is a global public health crisis that is yet to be contained. Effective management of HIV drug resistance (HIVDR) supported by close resistance monitoring is essential in achieving the WHO 95-95-95 targets, aiming to end the AIDS epidemic by 2030. Point-of-care tests (POCT) enable decentralized HIVDR testing with a short turnaround time and minimal instrumental requirement, allowing timely initiation of effective antiretroviral therapy (ART) and regimen adjustment as needed. HIVDR POCT is of particular significance in an era when ART access is scaling up at a global level and enhanced HIVDR monitoring is urgently needed, especially for low-to-middle-income countries. This article provides an overview of the currently available technologies that have been applied or potentially used in HIVDR POCT. It may also benefit the continued research and development efforts toward more innovative HIVDR diagnostics.

## 1. Introduction

The HIV/AIDS epidemic has spread to all populated continents in the past four decades, with no sign of ending in the foreseeable future. HIV has infected 79.3 million people since it was identified in the early 1980s, and approximately 36.3 million people have died from AIDS-related illnesses thus far [1]. In 2014, UNAIDS declared ambitious new targets (95-95-95) to end the HIV epidemic by 2030 [2,3]. However, a challenge that hinders the elimination of HIV is its ability to constantly mutate genetically and antigenically [4]. The high variability of HIV causes the emergence of drug-resistant variants, reducing the effectiveness of available antiretroviral (ARV) drugs [4,5]. As access to antiretroviral therapy continues to scale up globally, HIV drug resistance (HIVDR) has become an imminent danger that threatens the substantial strides taken by UNAIDS and impairs the maximization of antiretroviral therapy (ART) benefits [5].

HIV infections are treated with drugs that target viral proteins essential for their replication, such as protease (PR), reverse transcriptase (RT), and integrase (IN). Nucleoside and non-nucleoside reverse transcriptase inhibitors (NRTI and NNRTIs) prevent the reverse transcription of HIV RNA to proviral DNA. In contrast, protease inhibitors (PIs) prevent the cleavage of HIV polyproteins, and integrase inhibitors (INIs) interrupt viral integration into the host genome. The rise of HIV drug resistance mutations (HIVDRMs) may render these drugs inefficient in virological suppression for all available ART agents. As such, genotypic HIVDR typing aims to examine the presence of known HIVDRMs, qualitatively or semi-quantitatively [6]. HIV RNA and proviral DNA represents replication-competent viruses and archived/historical viral populations respectively. Therefore, the detection of HIVDRM(s) in HIV RNA and DNA may have different clinical application values. For instance, HIVDRMs in HIV DNA may only inform the treatment initiation with proper ARV drugs, while HIVDRM detection in HIV RNA benefits both ART initiation and subsequent regimen adjustment.

To minimize the impacts of HIVDR variants, the WHO recommends routine surveillance of HIVDR to monitor ART and pre-exposure prophylaxis (PrEP) distribution [4]. The information obtained can also be facilitated by countries when forming their national treatment guidelines to optimize patient outcomes [4]. Since most infections occur in the developing world, an ideal HIVDR assay should be accountable and readily accessible/operable in resource-limited settings (RLS) [7]. Point-of-care tests (POCT) are vital for de-centralized HIVDR monitoring, which offers lower testing costs, broader test access, shorter turnaround time, and timely initiation of effective ARV treatment and regimen adjustment as needed [8]. In addition, an ideal POCT should meet the ASSURED (**A**ffordable, **S**ensitive, **S**pecific, **U**ser-friendly, **R**apid, **E**quipment-free, and **D**eliverable) criteria endorsed by WHO (Geneva, Switzerland) [7].

POCT has been of great interest to HIVDR professionals for decades. It is acknowledged that the topic of HIVDR POCT had previously been reviewed by others [8,9,10]. While minimizing the overlap with the previous literature, we focus in this article on the recent advances in the previously examined POCTs, newly emerged technologies that have recently been attempted for HIVDR and also those assays with great POCT potentials but yet to be validated for HIVDR.

## 2. Technologies Attempted for HIVDR POCT

Conventional genotypic HIVDR typing relies on Sanger sequencing of target HIV genes and examines the presence of all known HIVDRMs within collectively. In contrast, while the mechanisms vary, all attempted HIVDR POCTs thus far target single or multiple selected known HIVDRMs only. Described below are several near-POCTs attempted for HIVDR testing thus far.

### 2.1. Oligonucleotide Ligation Assays (OLA)

OLA is a point mutation test initially developed by Landegren et al. to detect mutations associated with sickle cell anemia [11,12]. The assay was devised based on the premise that two adjacent oligonucleotide probes hybridized to a specific DNA sequence could be covalently bonded with a ligase that will discriminate against mismatched bases [11,13]. Frenkel et al. modified the OLA to detect HIVDR mutations in the HIV-1 *pol* gene with colorimetry or spectrophotometry [14,15,16]. OLA is the most-studied POCT for HIVDR. It has been implemented to detect HIVDR mutations associated with NNRTIs and NRTIs in Thailand, Zimbabwe, and Kenya [17,18,19,20]. Panpradist et al. recognized the need to improve the detection step, which proved too extensive and complex [12]. As a result, they created the OLA-Simple, allowing ligated products to be viewed as colored lines on a lateral flow strip either with a scanner or by plain sight [12,21,22].

**Figure 1:**
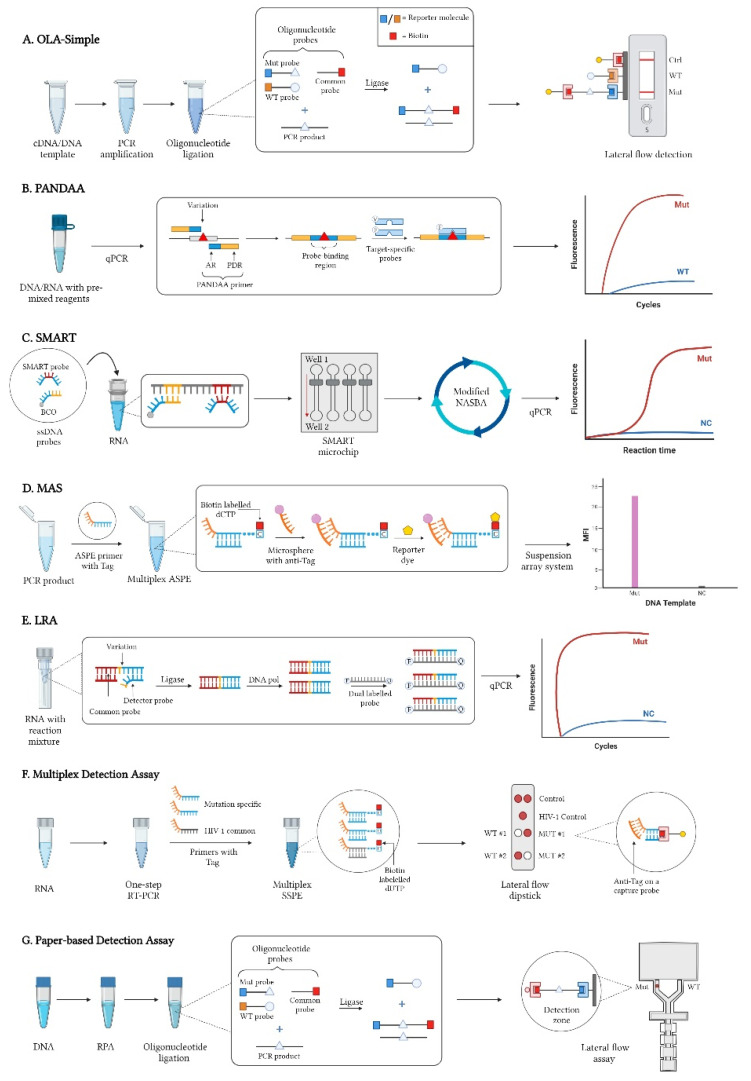
**Simplified workflow of the exemplar POCTs**. (**A**) In the OLA-Simple workflow, using pre-made dry reagents, RNA is used to make PCR products that will undergo oligonucleotide ligation. In this step, mutant (Mut)/wild-type (WT) probes with a reporter molecule will bind adjacent to a common probe with biotin to a complementary sequence on the template. The ligation products are eventually visualized using a lateral flow device; (**B**) PANDAA workflow, displaying how primers and probes bind to their specific target to determine the mutation of interest. Starting with the binding of PANDAA primers, qPCR will generate a homogenous population with a probe-binding region, followed by the annealing of a target-specific probe with FAM fluorophore (F) to detect the single nucleotide polymorphism. Wild-type specific probes are labelled with VIC fluorophore (V) for comparison. (**C**) The SMART assay combines molecular biology with microfluidics. The ssDNA probes are first added to RNA, where the SMART probe will bind to the mutation sequence and the BCO binds to a conserved sequence. Next, a SMART microchip will facilitate the separation of bound and unbound probes from well 1 to 2. This step is followed by a modified NASBA that will amplify probes and generate a sequence for a molecular beacon to identify the presence or absence of a mutation. (NC = negative control). (**D**) The MAS assay utilizes ASPE primers labelled with Tag to discriminate against a mutation. Primers are first added to a PCR product, and multiplex ASPE ensues. While amplification proceeds, biotin is incorporated into the final product and the Tag/anti-Tag sequences will bind to one another. After, a reporter dye will find biotin and detect hybridized products using a suspension array system. Data is then recorded by measuring the mean fluorescence intensity (MFI). (**E**) The LRA assay starts with adding RNA template to a reaction mixture containing ligases, DNA polymerases and oligonucleotide primers. Ligation occurs between a common probe that is complementary to the RNA template and a detector probe that is complementary to the variant. DNA polymerase will then become activated, and qPCR will amplify ligated products using dual-labelled probes with fluorophore (F) and quencher (Q) for detection. (**F**) Multiplex detection assay that uses specific primers with a Tag sequence and a lateral flow dipstick to detect mutations. PCR samples undergo a multiplex SSPE, and biotin is incorporated into the extended products. As amplicons flow through the dipstick, they bind to a complementary anti-Tag and anti-biotin antibodies with gold nanoparticles will produce red dots for identification. (**G**) A paper-based assay that combines different techniques to detect HIVDRM. It starts with RPA, followed by oligonucleotide ligation at the site of interest. Products are then applied to an ELISA lateral flow assay, where fixed antibodies will hybridize with reporter molecules. Then streptavidin conjugated with horseradish peroxidase binds to biotin to produce brown precipitates for signal detection.

The latest version of the OLA-Simple is capable of detecting HIVDRMs across multiple HIV-1 subtypes (A, B, C, D, and CRF01_AE) using different specimen types (dried blood spots, peripheral blood mononuclear cells, and plasma) [21]. There are four main steps involved in OLA-Simple, as illustrated in Figure 1A: (1) acquirement of a cDNA/DNA template, (2) PCR amplification, (3) ligation of oligonucleotide probes that identify single mutations, and (4) lateral flow detection [12,22]. In the ligation step, a genotype (mutation or wild-type) specific probe coupled with a reporter molecule and a common probe with biotin will bind adjacent to one another to a complementary sequence on the template [14,15]. The ligated products are then captured with immobilized antibodies on the lateral flow strip and detected with anti-biotin antibodies conjugated with gold nanoparticles to generate lines on the strip [21].

OLA-Simple has been successfully applied in detecting HIVDRMs across multiple major HIV-1 subtypes using specimens from Kenya, South Africa, Peru, Thailand, and Mexico. High concordances were obtained between the results from OLA-Simple and those from Sanger sequencing and even high-sensitivity HIVDR assays such as Next Generation Sequencing (NGS) with a mutation frequency cut-off at 1% [21,22]. The significant advantages of this assay include the use of lyophilized reagents for fast and accurate setup and the elimination of purification between steps [21,22]. The major equipment required to complete this assay includes a thermocycler and an office scanner linked to instructional software [21].

OLA-Simple is the best-developed near-POCT HIVDR thus far that has been validated for multiple key HIVDRMs from different HIV-1 subtypes. The instructional software by the assay developers also ensures the user-friendliness of performing OLA-Simple assay by inexperienced lab personnel, especially in RLS. Despite the promising approach of the OLA-Simple, it needs extra machinery to generate a DNA template, electricity to operate, and storage for lyophilized reagents. Proper training may also be necessary to avoid cross-contamination between different steps.

### 2.2. Pan-Degenerate Amplification and Adaptation (PANDAA)

Allele-specific PCR is one of the most widely used tests for HIVDR identification using quantitative PCR (qPCR) and is one of the many foundations of current POCTs. PANDAA is a point mutation assay developed by MacLeod et al. to tolerate the diversity of nucleotide sequences flanking the target mutation site, which showed a 96.9% sensitivity and 97.5% specificity for quantifying HIVDRMs present at ≥5% [23]. In a traditional qPCR reaction, the binding of probes that are not in perfect complementation to the template are unstable and can produce false-negative results. In contrast, the PANDAA assay addresses high sequence variability through normalization of probe-binding regions, as seen in Figure 1B. PANDAA primers have two main features: (1) a pan-degenerate region (PDR) containing degenerate bases to account for nucleotide variability and (2) an adaptor region (AR) that matches the probe-binding regions flanking the mutation of interest [24].

PANDAA requires no separate cDNA synthesis or PCR procedures, and the starting materials could be RNA or DNA. During the initial qPCR cycles, site-directed mutagenesis will occur to generate a population of homogenous amplicons with similar probe-binding regions complementary to the probes. This step removes any secondary polymorphisms that interfere with probe hybridization in a traditional qPCR. A target-specific probe will bind in the next stage, and qPCR results will differentiate between a mutant and a wild-type. While these primers were created by combining multiple HIV-1 subtypes to build a consensus sequence, the PANDAA assay can be curated to accommodate local HIV-1 sequence diversity [23]. One intrinsic limitation of PANDAA, and other allele-specific assays in general, is that a negative readout from it implies the absence of the target DRM allele. It could result from wild-type template, shown by a positive outcome from the wild-type control, or from a new allele at the target locus, which may render negative results for both wild-type and mutation-specific reactions.

Kouamou et al. assessed the diagnostic accuracy of PANDAA against plasma samples from patients in Zimbabwe that have acquired HIVDR [25]. Five HIVDRMs associated with resistance against NNRTIs and NRTIs were examined with PANDAA, and the results were compared against data from Sanger sequencing. The results demonstrated that PANDAA rendered excellent sensitivity (95~98%) and specificity (83~100%), although the readouts fluctuated among assays targeting different HIVDRMs. Maraupala et al. conducted another study to test the diagnostic accuracy of PANDAA against Sanger in a cohort of patients from Botswana to use the assay as an alternative approach for rapid HIVDR test, and high concordance was observed between the data obtained from the two compared assays [24]. This positions PANDAA as a promising assay for HIVDR, although further refinement is required to meet the ASSURED criteria [7].

PANDAA requires a qPCR machine, but there is no need for bioinformatics support in data interpretation [26]. One significant advantage of PANDAA is that it mitigates the impacts of sequence diversity in the flanking region, which would inevitably affect the probe binding and reduce assay sensitivity and accuracy. Still, it requires either RNA/DNA as a starting material, which indicates an extra step on top of the assay.

### 2.3. SMART (Simple Method for Amplifying RNA Targets)

The prevalence of influenza prompted McCalla et al. to develop a method to amplify RNA with engineered ssDNA probes [27]. They reasoned that the availability of rapid POCT diagnostics would aid the healthcare community in containing known infections and preventing antiviral misuse. Their research found nucleic acid sequence-based amplification (NASBA) assays advanced with the incorporation of microfluidic devices showed a positive response. McCalla’s methodology realized the benefit of this combination and made crucial modifications to NASBA to remove RNA secondary structures that hindered the assay and presented the SMART assay. Morabito et al. then repurposed the assay to detect HIVDR mutation from HIV-1 samples [28].

The SMART technique uses two ssDNA probes that will bind to a specific RNA target sequence: (1) biotinylated capture oligonucleotide (BCO) attached to a streptavidin-coated magnetic bead (SMB) binds to a conserved region and (2) a SMART probe binds to the mutated region (Figure 1C). The two probes are added to the solution to bind to the RNA target. The solution is then added to the SMART microchip, where it will pass through a microfluidic channel from one well to the other while a magnet separates bound and unbound structures. Afterwards, the modified NASBA will isothermally amplify the probes and molecular beacons will quantify data in real-time [27,28,29].

In this assay, amplification and detection rely on the specific hybridization of the SMART probe rather than the target RNA. The SMART probe can be engineered to have favourable or unfavourable binding energies to ensure it does not bind to other oligonucleotides. Additional benefits are the use of microfluidics, which provide a close, efficient, automated system that reduces hands-on time, human error, and probable contamination. To complete this assay, a microfluidic device and a qPCR are the main components needed [27,28,29]. Limitations in this assay consist of the absence of an extraction step to obtain an RNA template and qPCR, which utilizes molecular beacons. Concerns may also arise as to proper laboratory operation and the requirement of technicians to be trained. Again, a laboratory space will be essential in implementing this method. Moreover, this preliminary study has only tested a single NNRTI mutation, and the lack of real-world studies hinders the determination of feasibility of the assay, although it holds great promise for POCT application.

### 2.4. Multiplex Allele-Specific (MAS) Assay

This assay was developed by Zhang et al. to address issues with existing POCTs only detecting one or few mutations per test. Based on a suspension array technology, they produced the MAS assay that would allow simultaneous detection of multiple major HIVDRs [30,31,32,33]. In their study, allele-specific primer extension (ASPE) primers were designed to target HIVDRMs associated with NNRTIs, NRTIs, and PIs in HIV-1 subtype C [31,33]. The ASPE primers are further designed so that the 3′ end contains an allele-specific nucleotide, while its 5′ end has a Tag sequence [31].

The assay begins with adding ASPE primers to a reaction tube with a reagent mixture and the templates (Figure 1D). Primer extension occurs and biotin-labelled dCTP is incorporated into the derived amplicon. Afterwards, hybridization occurs where microspheres containing an anti-Tag anneal to the extended DNA fragment through complementary Tag sequence. A reporter molecule will then bind to the biotin and record the mean fluorescence intensity of each microsphere based on its unique internal dye.

In a follow-up study, Zhang et al. adapted the assay for subtype B by altering ASPE primers and applied it to dried blood spot specimens collected from patients on antiretroviral therapy [33]. For both HIV-1 subtypes B and C, MAS assays showed high concordance and comparability when compared to conventional Sanger sequencing [31,33]. The flexibility of the suspension array technology allows MAS assay to be easily adapted to create any ASPE primer and corresponding microspheres. Unlike sequencing, the results can be easily interpreted and reported right away [31]. The sensitivity of MAS assay ranged from 1.56% to 12.5% depending on the HIVDRM being examined [33].

The extreme variation among HIV-1 sequences warrants the need for specific primers to be made. In addition, as a PCR product is the starting material, raw material will have to be processed to obtain a template. There is also the requirement for a suspension array system to perform the assay and, as previously mentioned, such equipment comes with an extra burden. Considering the logistic and operational constraints as the assay is now, more development efforts are warranted to apply MAS in practice for HIVDR POCT.

### 2.5. Ligation on RNA Amplification (LRA)

A preliminary study by Barany exhibited a ligase-mediated detection technique to distinguish between mismatched and complementary bases [34]. This process joins two oligonucleotides together when they bind to a complementary sequence on a template. The products then undergo cyclic amplification with another set of oligos complementary to the original ones. In the presence of a mismatch, ligation and amplification are inhibited, suggesting the presence of a variation. This method has been adapted to anneal two DNA probes using miRNA as a template [35,36,37]. Zhang et al. noted the valuable role this assay played and the part it could have in HIVDR testing. After some modifications, one being the exclusion of cDNA production, which eliminated the risk of nucleotide misincorporation, the LRA assay was formed [38].

The LRA assay is a one-step, single-buffer scheme to detect point mutations from RNA. In a single tube with optimized buffer, ligase, hot-start DNA polymerase, and oligonucleotide primers are added (Figure 1E). The reaction has three phases: (1) ligation, (2) polymerase activation, and (3) quantitative PCR. The temperature is set low during the first stage, allowing ligase to be the only active enzyme. In this step, a common probe that fully complements the RNA target and a detector probe that is complementary only to the variant are hybridized adjacent to one another by a ligase. In the next stage, the ligase enzyme is inactivated and DNA polymerase is activated instead, signalling the start of the amplification phase. Ligated probes are then amplified with dual-labelled probes during qPCR for detection [38].

This method separates the ligation and qPCR reaction by exploiting hot-start polymerases. By doing so, only one reaction is active at a time, thereby achieving maximum sensitivity, which for K103N was determined as 1%. The results showed that this assay outperformed allele-specific PCR and pyrosequencing in detecting mutant specificity [38]. To perform this assay, a qPCR is needed, suggesting that it needs the minimum requirements of a laboratory.

Zhang et al. had only presented a proof of principle for this assay. The RNA template used had mutations introduced into the pol gene of the HIV-1 genome through site-directed mutagenesis. Further follow-up study and validation of this assay to examine different HIV-1 subtypes and HIVDRMs other than the K103N mutation they studied remain to be completed and are necessary before its potential for HIVDR POCT can be better assessed.

### 2.6. Multiplex Detection Assay

Gomez et al. first developed a novel method for rapid genotyping of blood groups using a lateral flow biosensor to prevent alloimmunization, a major complication during blood transfusions [39]. Using whole blood sample, multiplex PCR was performed, and amplicons were transferred onto a lateral flow strip. Products were then captured with probes and red dots appeared for blood group deduction. Combining strategies that were previously used for genetic diseases and cancer, they adapted the assay to detect HIVDRM [40,41,42].

The study executed a proof-of-concept test on HIV-1 subtype B plasma specimens to rapidly detect mutations that cause resistance in NNRTIs. The assay has two major components: (1) a rapid multiplex detection system utilizing sequence-specific primer extension (SSPE) primers containing a Tag sequence and (2) a dry reagent lateral flow dipstick that generates red dots (Figure 1F). As the sample migrates on the dipstick, the products are captured by probes with an anti-Tag sequence. Then, an anti-biotin antibody conjugated with gold nanoparticles will cause the generation of red dots for easy visual recognition. On the membrane, wild-types can be detected on the left, while mutants can be found on the right [43]. This assay was shown to have a limit of detection of 100 copies of plasmid DNA, while its sensitivity for mutation detection was determined as 10~20% depending on the HIVDRMs being examined. Notably, these findings remain to be validated using clinical specimens.

Besides the OLA-Simple, Multiplex Detection Assay is another methodology claimed to be near-point-of-care assay [43]. It proves to be versatile as primers and probes of this assay can be tailored to detect known or new HIVDR mutations prevalent in an area. It is specific, and no cross-reaction has occurred between sequences from different subtypes or between wild-type and variant sequences. Compared to Sanger, which has a lower detection limit of 200 copies per assay, the study achieved a lower detection limit of 20 copies per assay, demonstrating higher sensitivity. To perform this assay, some of the instruments required include a thermal cycler and a drying oven. Regardless of the positive indications of this assay, an extraction step is needed. Also, much like the LRA, it fails to provide much real-life evidence to showcase its practicality.

### 2.7. Paper-Based Detection Assay

This proof-of-concept assay builds on multiple techniques and previous work done by researchers. Bui et al. found success when they joined PCR with OLA to detect drug resistance in *Streptococcus pneumonia*. As discussed earlier, Panpradist et al. made significant strides in modifying the OLA-Simple [21,44]. Based on these findings, Natoli et al. established a technique that would isothermally amplify HIV products and detect drug resistance through a lateral flow [45].

The assay starts with using recombinase polymerase amplification (RPA) to amplify a portion of the HIV-1 pol region (Figure 1G). Afterwards, an oligonucleotide assay, akin to the OLA-Simple, is used to discriminate against wild-type, and visualization occurs through the paper-based ELISA. In the lateral flow membrane, antibodies corresponding to reporter molecules are immobilized on each side of the fork and will each capture ligated products as they flow through the membrane. Brown precipitates will then appear if ligated products are present. The detection limit of this assay was determined as 10^3^ copies of pre-amplification template while mutant templates were present at 20% [45].

RPA not only isothermally amplifies DNA in a short time, but it also tolerates impure samples, which is favorable in places where contamination is unavoidable. Reagents of this amplification technique can also be in lyophilized form, shortening preparation time. Additionally, the assay design proves to be specific as the fork in the membrane limits the aggregation of OLA products. To complete this assay, a heat block and a tabletop centrifuge is necessary for the RPA step prior to proceeding to OLA [45].

Although this assay can merely detect one mutation per test, it can be adapted to detect other high-impact mutations. At its current state, the assay is yet to have sample preparation as an integrated step, and more hands-on time is required when adding ELISA reagents to the membrane. Furthermore, gBlocks stocks were used as DNA template and the membrane design lacks a control region, lowering the validity of this assay. More research is needed to advance this technology towards POCT.

*HIV patients often elude clinics after a one-time visit because of stigma, inconvenience, travel costs, or other socio-economic factors. It is then essential for physicians to diagnose and treat the patients on the same day. POCTs for HIVDR will aid physicians when determining the best drug regimen to start with or switch to in order to achieve suppressed viral loads. Table 1 provides an overview of some general features and requirements of these assays. The estimates of the assay costs and assay time was excluded as these numbers can vary depending on manufacturers and number of samples, respectively.*

## 3. Other Potential POCT Technologies

As new methods arise, so do more opportunities for creating an ideal test for RLS. In this section, we explore two new technologies with the potential for simplified HIVDR typing.

### 3.1. Multiplex Solid-Phase Melt Curve Analysis

This melt curve analysis platform is a genotypic resistance assay that measures the hybridization, capture, and dissociation of multiple nucleic acid targets to and from surface-bound oligonucleotide probes. The probes have two parts: (1) a complementary sequence to HIV-1 strains and (2) a nucleotide triplet complementary to a codon at a drug resistance mutation. First, fluorescently labelled DNA is added to the oligonucleotide microarray at one temperature (i.e., 55 °C). Then, the concentration of labelled DNA captured is monitored in real-time by the probes as the solution temperature progressively increases. A time-series data is generated that defines duplex stability, which is used to identify the correct codon at the position of a drug resistance mutation. In vitro experiments were performed with HIV-1 plasma samples and culture supernatants, containing HIV-1 subtype A, B, C, D, CRF01_AE, and CRF02_AG [46]. Although this is a promising approach, operation in a closed-tube format and removing the wash step are essential. Multiplex PCR will need to be executed to detect the entire set of known HIVDRMs. Lastly, a production version of a prototype chip was used to detect fluorescence, so it is unsure how this would translate in real-world settings [46].

### 3.2. µBAR Platform

Myers et al. have developed the Microfluidic Biomolecular Amplification Reader (µBAR) in an attempt to combine electronics, optics, microfluidics, and molecular biology. The µBAR is a battery-powered, portable instrument capable of isothermal amplification of multiple markers with the use disposable microfluidic assay cartridges. First, the sample (i.e., blood, sputum, or saliva) is loaded onto a disposable microfluidic cartridge, where the system uses a loop-mediated isothermal amplification (LAMP) technique. The cartridge is then inserted into the µBAR, where it will control assay temperature, illuminate the chip, and monitor real-time fluorescence signals from individual reaction chambers [47,48].

Previously, the LAMP assay was verified to detect HIV and malaria from blood samples and TB drug resistance from sputum samples. Myers et al. have also exhibited use of the LAMP assay on the µBAR platform in detecting the HIV integrase gene. The platform also has GPS and cellphone connectivity for healthcare delivery in remote locations and epidemiological surveillance. The chip contains six inlets, meaning multiple samples can be loaded simultaneously. In its current form, the µBAR requires more work to modify for HIVDR detection [48].

### 3.3. Oxford Nanopore MinION (ONT) Sequencing

The increase in using NGS technologies to detect HIVDR have been on the rise. It is noteworthy that most of available NGS platforms are not even close to the bedside POCT considering their prohibitive instrument and reagent costs, demanding technical operation and complexity in data interpretation. One exception could be the MinION platform, developed by the Oxford Nanopore Technologies (https://nanoporetech.com/, accessed on 16 June 2022). MinION is thus far the only portable device that execute NGS on DNA or RNA templates with minimal requirement for additional instrumental and technical support.

Gonzalez et al. pioneered applying MinION sequencing to HIVDR analysis. HIV RNA was first extracted from plasma samples followed by PCR amplification. PCR products were then prepared for MinION library preparation and a sequencing library was generated to load into a flow cell. Good concordance was observed between the MinION consensus sequences and the Sanger sequencing outputs from the same patients, regarding both the sequence identity and HIVDR profiling [49]. While their findings support the usage of ONT in decentralized laboratories, the scarcity of supporting data from other labs warrants further investigation on the full potentials of MinION technologies in HIVDR POCT.

*The technologies listed in this section are but a snippet of potential and relevant assays. For example, techniques that use GeneXpert, Clustered Regularly Interspaced Short Palindromic Repeats (CRISPR), and High-Resolution Melting (HRM) have also been implemented in drug resistance detection for varying pathogens. Perhaps these methods, coupled with the rise in technological advancement, may inspire new HIVDR POCTs.*

## 4. Conclusions

The expansion of ART access comes with a growing concern for the rise in HIVDR. HIVDR monitoring is essential for effective HIV/AIDS management at individual and population levels. Conventional Sanger sequencing-based HIVDR genotyping may not be readily assessable, especially in RLS, for logistical and operational reasons. POCT offers a quick and affordable solution administered at or near patient care. The assays explored here show the progression of each test, where they stand, and adjustments that need to be made. Although the work that has been done is impressive, no such assay entirely embodies the ASSURED criteria. A fully validated POCT that satisfies the set standards and meets all the needs for HIVDR diagnostics, especially in RLS, has yet to be developed. More research still needs to be done as POCTs are indispensable in controlling the spread of drug-resistant HIV.

## Figures and Tables

**Table 1:** An overview of current technologies promising for HIVDR POCT.

POCTs	Starting Material	Subtype Specificity	Major Equipment Required	Validated against Sanger	Refs.
OLA-Simple	DNA or RNA	HIV-1 (A, B, C, D, AE)	Thermocycler, Office scanner	✓	[16]
PANDAA	DNA or RNA	Subtype independent	qPCR machine	✓	[23]
SMART	RNA	HIV-1 (any subtype with a K103N region)	Microfluidic device, qPCR machine	〤	[27]
MAS	DNA	HIV-1 (B, C)	Compact suspension array system	✓	[31,33]
LRA	RNA	HIV-1 (any subtype with a K103N region)	qPCR machine	〤	[38]
Multiplex Detection Assay	RNA	HIV-1 (B)	Thermocycler,	✓	[43]
			Dry oven		
Paper-based Detection Assay	DNA	HIV-1 (B)	Heat block, Thermocycler, Tabletop centrifuge	〤	[45]
